# Medical and Physical Intelligence

**Published:** 1805-06-01

**Authors:** 


### MEDICAL AND PHYSICAL INTELLIGENCE.

[ FOREIGN AND DOMESTIC. ]

Dr. Lind, in a Letter to Dr. Batty, dated Windsor, 9th April, 1805, writes as follows:

" Dear Sir,

" The enclosed was sent from Bombay, by my brother-in-law, lieutenant colonel Mealy, as a curious and authentic piece of intelligence respecting the cow-pox. If it proves of any use to you, I shall esteem myself happy in having had it in my power to communicate it to you.—If you should meet Mr. Mac Gregor, Surgeon to the Royal Regiment of Horse Guards, who is at present in London, and sometimes visits Sir Joseph Banks, he knows Mirza Mehedy Ali Khan, and can give you information respecting the cow-pox at Bombay."

*Translation of a written Memorandum from the* Nawaub Mirza Mehedy Ali Khan, *now at Bombay.*

During the period of my abode in the district of Benares, my eldest son being taken ill of a bad kind of the small-pox; and my friends interesting themselves for my comfort and his relief, one of them, named Slookum Chund, a Hindoo, pointed out to me that there was in the city of Benares, one Alep Choby, a Brahmin from Owd, whose practice was chiefly confined to this malady. Him I therefore lost no time in sending for to the town of Ghazeepoor, where I dwelt, and he arrived on the ninth day of the eruption. On seeing which he observed, that if the eruption had not taken place he would have endeavoured to facilitate and render it easy; but that now it was too late. On asking Choby what his process was? he said, " From the matter of the pustule on the Cow, I keep a thread drenched, which enables me at pleasure to cause an easy eruption on any child; adoring at the same time Bhowanny, (who is otherwise called Debee, Mata, and Seetla, and who has the direction of this malady) as well in my own person as by causing the father of the child to perform the like ceremonies; after which I run the drenched string into a needle; and, drawing it through between the skin and flesh of the child's upper arm, leave it there; performing the same operation in both arms, which always ensures an easy eruption.—On the first appearance of which, the child's father, or guardian, renews his worship to Bhowanny; and as the animal this Goddess rides on is an ass, it is customary for such parent, or guardian, to fill his lap with grain, which an ass is sent for to eat up; which observances ensure the propitious direction of Bhowanny, so that only a very few pustules make their appearance; nor does any one die under this process!" Thus far did I learn from Alep Choby.

Upon

Upon referring on this subject to a native, well-versed in the learning and customs of the Hindoos, he told me that the practice thus described by Choby was not general among them; but confined to those who where attached to the worship of BHOWANNY, and adored her with implicit faith—and upon my asking this person, whether he was aware how the matter of the pustule was got from the cow, and whether all cows had such pustules, or only those of a certain description? He answered, that on these points he possessed no information; but had certainly understood that the cows had these pustules break out upon them, and that from the matter thereof children were infected; acknowledging, however, that he spoke not this much from ocular knowledge but from report.

---

A few months since a female child was born in New-York, America, whose ureters, instead of terminating in the bladder, according to the usual course of nature, discharged the urine externally a little above the pubes. In this monstrous conformation the mons veneris seemed to be wanting, and a reddish carnosity to occupy its place. Through this there were two orifices, corresponding to the two ureters. The urine constantly trickled from them, except when, after a time of rest, the child suddenly struggled or cried, and then it would spirt forth from both openings in a continual stream. The presumption, of course, is, that the bladder is wholly wanting. The genital parts partake somewhat of this odd configuration; and the umbilicus is considerably lower, and the anus further forward than common. In other respects the infant hitherto seems to be well formed, and in good health.

---

Mr. MANNING, sculptor, No. 14, Warren-street, Fitzroy-square, London, formerly pupil to the late Mr. Bacon, has modelled from the life a bust of Dr. Jenner, which is now in the Exhibition of the Royal Academy, Somerset House. This model, which has been honoured with the approbation of the best judges, and is allowed to be an admirable likeness of Dr. Jenner, he intends to publish. The terms are three guineas; one half to be paid at the time of subscribing, the other half on the delivery of the bust. The casts will be delivered in rotation, as they are subscribed for.

---

Dr. ADAMS, author of Morbid Poisons, is unanimously appointed to succeed Dr. Woodville as physician to the Small-Pox and Inoculation Hospitals. We rejoice that a person so well qualified for the situation, and one who has always been so zealous a prometer of vaccination, is placed in a station where he can render so much service to the cause.

---

M. BIOT has succeeded in forming water from hydrogen and oxygen, by compression only, independently of the electric spark. The compression, by bringing the particles of gas into intimate union, makes them throw out a quantity of heat sufficient to set them on fire. Great caution is necessary in making the experiment.

---

IR

In a few weeks will be published a work, entitled, "Observations on the Simple Dysentery and its Combinations," containing a review of the most celebrated authors who have written on that subject, and also an investigation into the source of contagion in that and some other diseases. Previous to its publication a prospectus may be had gratis of all the medical booksellers. This interesting subject has, we understand, long engaged the attention of Dr. HARTY.

---

An Enquiry into the Nature of the Disease which so lately prevailed at Gibraltar; with Remarks on Epidemic Fevers in general, and on the recent proceedings of the Society for bettering the condition of the Poor relative to the prevention of Contagious Malignant Fevers in the Metropolis, is preparing for the press, by SEGUIN HENRY JACKSON, M. D. &c. &c.

---

Mr. PARKINSON, of Hoxton, has a work in the press, on the Gout, in which he purposes to consider of the modes recommended for the cure of this disease, and to offer some remarks on its nature and origin.

---

Dr. REID, of Grenville Street, Brunswick Square, will recommence his Lectures on the Theory and Practice of Medicine, on Tuesday the 25th of June.

